# Hitting the Gym with Fit3D: Benchmarking and Improving Monocular 3D Human Reconstruction on Extreme Fitness Motions

**DOI:** 10.3390/jimaging12070311

**Published:** 2026-07-09

**Authors:** Mihai Fieraru

**Affiliations:** Institute of Mathematics of the Romanian Academy, 010702 Bucharest, Romania; mfieraru@imar.ro

**Keywords:** 3D human pose, 3D human shape, fitness, human reconstruction, motion capture, benchmark

## Abstract

Fitness motions present some of the most challenging cases for monocular 3D human reconstruction: extreme articulations, heavy self-occlusion, and frequent self-contact. The *Fit3D* dataset captures these motions at large scale via a 12-camera VICON motion capture system synchronized with 4 RGB cameras, but in its original release provides only 3D skeletons. This paper contributes three studies built on top of *Fit3D*. First, we design and validate a methodology for constructing dense GHUM and SMPL-X pseudo-ground-truth shape and pose annotations on top of the raw MoCap: an optimization-based fitting pipeline that combines markers, multi-view 2D keypoints, separate body and hand normalizing-flow priors, and a self-collision loss, which we show improves on the marker-only MoSh++ baseline on the hands and extremities. Second, we define a standardized evaluation protocol—metric set, frame sampling, and coordinate conventions—for monocular 3D human reconstruction on *Fit3D*, served through the IMAR-hosted Fit3D evaluation resource, and use it to conduct a comparative benchmark study of 19 representative methods spanning optimization-based, single-frame, and video-based families; the two trained on *Fit3D* (NLF and SMPLest-X) lead the position and orientation metrics, respectively. Third, a controlled fine-tuning experiment shows that adding *Fit3D* to the training mixture of a strong baseline (HMR2.0) sharply lowers error on the hardest fitness poses without degrading out-of-domain generalization. The *Fit3D* dataset and the GHUM/SMPL-X annotations are available, under a non-commercial research license, through the IMAR Fit3D resource; as of June 2026, 1088 academics have registered for access.

## 1. Introduction

Monocular 3D human pose and shape reconstruction has matured rapidly: methods such as 4DHumans [1], NLF [2], CameraHMR [3], SMPLest-X [4] and SAM 3D Body [5] now produce convincing meshes on standard benchmarks like 3DPW [6] and EMDB [7]. Fitness motions, however, remain a regime in which these methods struggle. Deep squats, lunges, bridges, handstands, kicks, and weight-loaded poses generate extreme articulations, severe self-occlusion, and frequent self-contact—failure modes that the dominant training corpora (Human3.6M [8], 3DPW [6], BEDLAM [9], AGORA [10]) do not adequately cover. Progress in this regime is further bottlenecked by two missing ingredients: there is no parametric (GHUM, SMPL-X) pseudo-ground-truth body annotation on a representative fitness corpus, and no standardized evaluation protocol that researchers can compare against.

The *Fit3D* dataset, introduced by Fieraru et al. [11], addresses the data side of the problem: it captures 13 subjects (one licensed fitness instructor and 12 trainees) performing 47 simple and compound exercises that cover all the major muscle groups, using a 12-camera VICON motion capture system synchronized with 4 calibrated RGB cameras, and contains approximately 3.0 million RGB frames together with the corresponding 3D MoCap skeletons and per-subject 3D body scans. However, in its original release, *Fit3D* provides only the 3D skeletons, which limits its direct use as supervision for modern parametric-body-model regressors.

**Figure 1:**
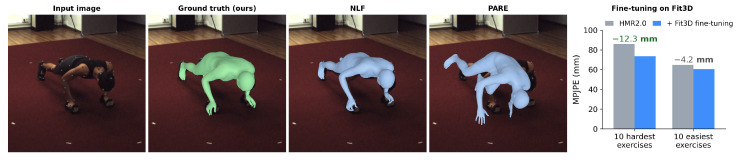
Overview of the contributions. From left: an input frame from the *Fit3D* test set (subject s02, *man maker*—a dynamic, ground-level compound exercise); the GHUM/SMPL-X pseudo-ground-truth annotation for the frame, obtained by the multi-view marker- and keypoint-driven optimization of Section 3.2; predictions of two of the 19 methods evaluated under our benchmark protocol (Section 4)—NLF, the strongest method, trained on *Fit3D*, and PARE, which fails on this pose and the effect of fine-tuning HMR2.0 with *Fit3D* supervision (Section 6), which lowers the error on the ten hardest exercises by 12.3 mm on average, versus 4.2 mm on the ten easiest. The two predicted meshes are rendered at a common root depth, as in the qualitative comparison of Section 5.

In this paper we make three contributions on top of the existing *Fit3D* dataset. First, we design and validate the methodology used to construct dense GHUM [12] and SMPL-X [13] pseudo-ground-truth shape and pose annotations on top of the raw MoCap, by an optimization-based fitting pipeline that combines VICON markers, multi-view 2D keypoints, separate body and hand normalizing-flow priors, and a self-collision loss in order to accommodate the extreme articulations and self-occlusions characteristic of fitness routines. These annotations are available through the IMAR Fit3D resource [11] and have since been adopted in the training corpora of large-scale 3D human reconstruction methods such as NLF [2] and SMPLest-X [4]. Second, we define a standardized evaluation protocol—metric set, frame sampling, and coordinate conventions—and conduct a comparative benchmark study of monocular 3D human reconstruction on *Fit3D*; the protocol is served through the IMAR-hosted Fit3D evaluation server [11] at https://fit3d.imar.ro/ (accessed on 5 July 2026), which accepts GHUM, SMPL-X, and 3D-joint submissions and reports a standardized set of metrics. Using it, we benchmark 19 representative methods spanning optimization-based, single-frame, and video-based families (2019–2026) and analyze the impact of including the dataset in their training corpus. Third, we run a controlled fine-tuning experiment that isolates the effect of *Fit3D* supervision, showing that it improves a strong baseline most on exactly the hard, out-of-distribution poses where current methods fail, without regressing out-of-domain performance. Figure 1 gives an overview of the three contributions.

## 2. Related Work

Visual human sensing has been extensively studied [13,14,15,16,17,18,19,20,21,22,23,24,25,26]. Applications exist in many domains such as the automotive industry [27,28], fashion industry [29,30], activity recognition [31] and many others. One particular area that has been less considered by research is high-quality 3D human reconstruction for fitness, the focus of the present work.

3D Human Pose and Shape Datasets.

Large-scale 3D human datasets have been the main driver of monocular 3D human reconstruction. Human3.6M [8] captures daily activities of 11 subjects with a MoCap rig, MPI-INF-3DHP [18] extends the setting to outdoor sequences, and 3DPW [6] pioneers in-the-wild capture via IMUs. More recent datasets target specific failure modes: EMDB [7] provides accurate world-grounded motion via electromagnetic sensors, RICH [32] adds dense scene contact, MOYO [33] captures yoga and a wide range of extreme articulations, and BEDLAM [9] and AGORA [10] provide synthetic ground truth at scale. None of these datasets are specifically targeted at fitness training, where extreme articulation, weight loading, self-contact, and tight gym-style camera setups co-occur. *Fit3D* fills this gap by combining high-fidelity MoCap with parametric body-model annotations on a corpus of fitness exercises; Table 1 summarizes how it relates to these datasets along the axes of scale, capture modality, and the body-model ground truth available.

Monocular 3D Human Reconstruction.

Methods for monocular 3D human reconstruction range from optimization-based fitting (SMPLify-X [13]) to single-frame regressors that estimate SMPL, SMPL-X, or GHUM parameters from an image (PARE [34], HybrIK [35], PyMAF-X [36], CLIFF [37], 4DHumans/HMR2 [1], TokenHMR [38], Multi-HMR [39], CameraHMR [3], SMPLest-X [4], BLADE [40], NLF [2], SAM 3D Body [5]). Video-based regressors (WHAM [41], GVHMR [42], PromptHMR [43]) and refinement-style approaches (ScoreHMR [44]) additionally exploit temporal context or post-hoc consistency. We benchmark a representative cross-section of these families on *Fit3D* in Section 4.

Evaluation metrics and benchmarks.

Monocular 3D human reconstruction is most commonly scored with the mean per-joint position error (MPJPE) and its Procrustes-aligned form on Human3.6M [8] and 3DPW [6]. For parametric body models, these are complemented by the mean per-vertex error (MPVPE) on expressive SMPL-X benchmarks such as EHF [13] and RICH [32], and by the mean per-joint angle error (MPJAE) introduced with 3DPW [6], which measures orientation accuracy independently of limb length. To isolate body shape from pose, we additionally adopt the shape-only T-pose vertex error (MPVPE-T), following the PVE-T metric of Sengupta et al. [45]. Our protocol (Section 4) brings this metric set together on *Fit3D*, evaluating GHUM, SMPL-X, and 3D-joint predictions against the released annotations.

## 3. Fit3D

This section briefly summarizes the *Fit3D* dataset [11] that the present work builds on, and then describes the methodology we use to extend its annotations with dense GHUM and SMPL-X pseudo-ground-truth.

### 3.1. Dataset

The *Fit3D* dataset, introduced by Fieraru et al. [11], features 13 human subjects performing fitness exercises. Among them, there is one licensed fitness instructor (considered the reference for correctness in exercise execution), while the rest are considered trainees of various levels of skill.

The dataset was captured with a VICON motion capture system consisting of 12 motion cameras, synchronized with 4 RGB cameras. The capture process involved reflective markers affixed to either the subject’s skin or clothing. All subjects were dressed in gym-like attire that fits tightly on the body. During the recordings, the subjects used several typical gym objects: 2 dumbbells, a barbell, and a rubber band. A low-height, footless table was used to ease the difficulty of the exercises involving lifting the barbell.

The fitness exercises target the major body muscle groups: arms, legs, back, and abdomen. They are split into two groups: simple (involving basic repetitions such as *push-up*, *squat*, or *dumbbell biceps raise*) and compound (assuming more complex routines involving multiple body regions, such as *burpees*, entailing a push-up and a jump, or *clean and press*, assuming certain trajectories of the arms). Each subject was asked to perform each type of exercise for a minimum of 5 repetitions.

Subject heights range from 1.55 to 1.90 m and weights from 60 to 110 kg; the dataset covers a mix of fit subjects (persons who exert a high degree of physical activity) as well as less trained ones. *Fit3D* consists of approximately 3.0 million unique MoCap 3D skeletons synchronized with RGB images. Each subject was also 3D scanned. The dataset is split by subject into training (8 subjects), validation (2 subjects), and testing (3 subjects); the 10 training and validation subjects together comprise approximately 2.3 million images and the 3 test subjects approximately 0.7 million, with all exercise types present in every split. Unless noted otherwise, the marker-fitting comparison (Section 3.3) and the fine-tuning experiment (Section 6) use the 8 training subjects, while the benchmark (Section 4) evaluates on the 3 test subjects. Each video is also manually segmented into repetitions, with a total of 2964 annotated timestamps.

In its original release [11], *Fit3D* provides only the 3D skeletons synchronized with RGB. In Section 3.2, we describe the methodology used to augment each frame with dense GHUM [12] (and, by conversion, SMPL-X [13]) shape and pose pseudo-ground-truth (pGT), obtained by fitting the body model to the markers and multi-view RGB evidence. We use the term *pseudo-ground-truth* to emphasize that these annotations are produced by optimization-based fitting rather than by direct measurement, following the terminology adopted for comparable optimization-fitted datasets such as RICH [32]. Throughout, we reserve the unqualified term “ground truth” for the directly measured VICON marker positions, and refer to the optimized GHUM/SMPL-X parameters as pseudo-ground-truth or, where unambiguous, the *reference* annotations.

### 3.2. Motion and Shape Capture

By default, the VICON motion capture system is designed to track only the 3D position of markers attached to the body of a person and to regress a 3D skeleton underneath. Yet, we are interested in tracking the entire surface of the body, as well as the articulation of the two hands. To achieve our goals, we leverage any additional information that is available to us: scans of each subject, the four synchronized calibrated cameras, keypoint detectors, personalized marker placement on the body surface, pose priors, and self-intersection losses.

As fitness motions produce extreme articulations and significant self-occlusions, it can happen that the VICON system outputs erroneous marker positions or marker types. Therefore, we first manually fix these errors in the VICON Nexus software by switching marker identities and/or interpolating between correct tracks.

To capture the motion and shape of each subject, we use GHUM [12], a parametric 3D human body model. The model is learned from a large dataset of human shape and poses and is able to generate a mesh consisting of 10,168 vertices and 20,332 facets. Its inputs are body pose parameters θ represented as 6D rotations between the major limbs of the body (including hand articulations), body shape parameters β represented as the latent of a variational autoencoder, a global rotation R, and a global translation T. Estimating the motion and shape of a subject reduces to estimating the body shape parameter β from the 3D body scan, once per subject, and then optimizing θ, R, and T at each frame.

We first estimate the body shape β of each subject by fitting the GHUM body model to the point cloud generated by the 3D body scanner, following the methodology described in [12].

With the body shape β fixed, we focus on fitting the pose θ, global rotation R, and global translation T at each frame. The first frame of a sequence is initialized by running THUNDR [46] in each of the four RGB views and averaging the resulting GHUM parameters; subsequent frames are warm-started from the optimum of the previous one, exploiting temporal continuity of the motion.

We minimize a weighted sum of five losses. The marker alignment term

(1) $$L_{3D}=\frac{1}{K}\sum_{i=1}^{K}{∥m_{i}^{3D}-M_{i}^{3D}∥}_{2}^{2}$$

measures the squared error between the K=53 GHUM-regressed markers $m_{i}^{3D}$ and the corresponding (post-cleanup) VICON markers $M_{i}^{3D}$. Since the marker layout does not densely cover the hands, we additionally use a 2D keypoint alignment loss over the four synchronized RGB views,

(2) $$L_{2D}=\frac{1}{4J}\sum_{c=1}^{4}\sum_{n=1}^{J}{∥\pi_{c}\left(j_{n}^{3D}\right)-{\hat{k}}_{c,n}^{2D}∥}_{2}^{2},$$

where $j_{n}^{3D}$ is the *n*-th of the *J* joints regressed from the GHUM mesh, $\pi_{c}$ projects it into camera *c*, and ${\hat{k}}_{c,n}^{2D}$ are detections from a 2D keypoint predictor [47]. $L_{2D}$ acts primarily as a constraint on the hand joints, which are not covered by VICON markers.

Fitness motions induce large articulations and frequent self-occlusions; to keep the reconstruction physically plausible, we add a self-collision penalty. We approximate the GHUM mesh by a coarser watertight proxy mesh $\hat{M}$ with vertices $\hat{V}$, computed with the PHUM body model [48], and, following [48], apply the generalized winding number test of [49] to label each proxy vertex as inside ($L_{+}$) or outside the remaining body surface. The penalty

(3) $$L_{\mathrm{sc}}=\sum_{l\in L_{+}}{∥{\hat{V}}_{l}-\mathrm{NN}\left({\hat{V}}_{l},\hat{M}\right)∥}_{2}^{2}$$

pulls each inside vertex ${\hat{V}}_{l}$ toward its nearest neighbor $\mathrm{NN}({\hat{V}}_{l},\hat{M})$ on the outside of the proxy mesh. The body and hand poses are regularized by two separate normalizing-flow priors [50], ${\mathrm{NF}}_{b}$ for the body joints and ${\mathrm{NF}}_{h}$ for the hand articulation:

(4) $$L_{\theta }^{b}=∥{\mathrm{NF}}_{b}\left(\theta_{b}\right){∥}_{2}^{2},\;L_{\theta }^{h}={∥{\mathrm{NF}}_{h}\left(\theta_{h}\right)∥}_{2}^{2},$$

which penalize implausible articulations. Finally, a temporal smoothness term

(5) $$L_{s}=∥\theta^{t}-\theta^{t-1}{∥}_{2}^{2}+∥R^{t}-R^{t-1}{∥}_{2}^{2}+{∥T^{t}-T^{t-1}∥}_{2}^{2}$$

couples consecutive frames and damps jitter, where the pose θ and the global rotation R are differenced in their 6D rotation representation and T in metric coordinates.

**Figure 2:**
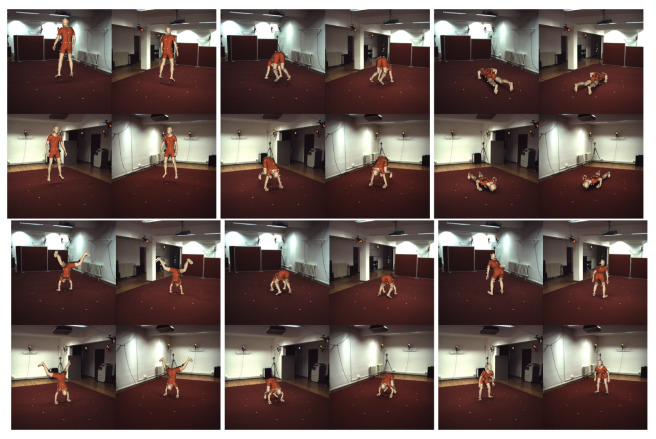
Motion and shape capture results. The fitted GHUM mesh is overlaid on each of the four synchronized RGB views (shown as a 2×2 grid per frame), for three frames of two exercise sequences: *burpees* (***top***) and *mule kick* (***bottom***). The latter illustrates the robustness of the fitting pipeline to extreme articulations and significant self-occlusions.

We minimize the weighted sum $L=\lambda_{3D}L_{3D}+\lambda_{2D}L_{2D}+\lambda_{\mathrm{sc}}L_{\mathrm{sc}}+\lambda_{\theta }^{b}L_{\theta }^{b}+\lambda_{\theta }^{h}L_{\theta }^{h}+\lambda_{s}L_{s}$ per frame with BFGS; the weights $\lambda_{•}$ were hand-tuned by visual inspection of the reconstructions. Some Fit3D motions lie outside the distribution the body prior was trained on—specifically *mule kick*, *warm-up 18*, and *warm-up 19*— causing $L_{\theta }^{b}$ to fight the data terms; for these three sequences, we lower $\lambda_{\theta }^{b}$ so the body regularizer does not bias the reconstruction away from the markers. Because marker placement on the skin varies by a few millimeters between subjects, we manually adjust each marker’s position on the GHUM template at two points: once per subject at the start of the capture, before optimization, and again whenever a marker detaches and is reattached, using a dedicated T-pose sequence recorded afterwards to re-register that marker. All reconstructions in *Fit3D* were visually inspected from multiple viewpoints during the development of this pipeline, and the loss list above is the outcome of that iterative refinement. Figure 2 shows example fits of the GHUM model, overlaid on the four synchronized RGB views, for two exercise sequences.

### 3.3. Comparison with MoSh++

**Figure 3:**
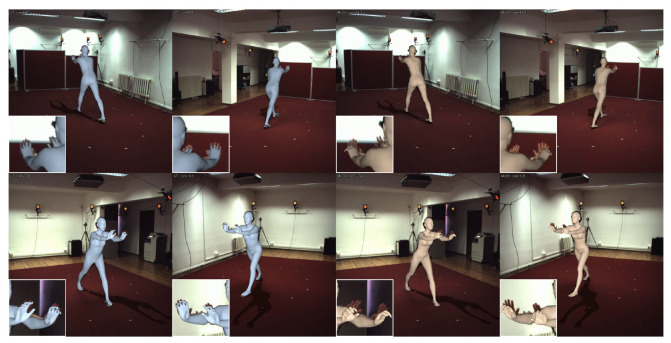
Hand articulation. Our multi-view motion and shape capture pipeline (*left two columns*) vs. MoSh++ [51] (*right two columns*), shown on a single frame seen from the four synchronized RGB cameras of our capture setup, arranged 2×2 per method. Our fits resolve realistic finger poses around the grip because $L_{2D}$ ties the GHUM hand joints to multi-view RGB keypoint detections; MoSh++ relies on VICON marker positions only and leaves the hands in a default rest pose. The bottom-left inset of each panel is a zoom on the corresponding pair of hands.

We also fit each frame with MoSh++ [51], the de facto standard for marker-driven body fitting, and find it insufficient for fitness motions: because MoSh++ consumes only VICON marker positions and ignores the synchronized RGB views, it has no signal on the fingers and leaves the hands in a default rest pose regardless of what the subject is gripping. Our pipeline addresses this gap directly through the multi-view 2D keypoint term $L_{2D}$, which propagates the visible finger configuration in the RGB streams into the GHUM hand joints. Figure 3 shows the contrast on a single frame seen from the four synchronized RGB cameras of our capture setup: our reconstruction (*left two columns*) versus MoSh++ (*right two columns*), with a zoomed inset on the hand region of each panel.

Quantitative comparison.

We quantify both effects on all eight *Fit3D* training subjects (Table 2). To measure hand articulation, we reproject the 21 hand joints of each fit into the four RGB views and compute their distance to the corresponding 2D hand keypoints from a strong whole-body keypoint detector [52], different from the 2D keypoint predictor used to fit the GHUM annotations (Section 3.2); aggregated across the four cameras, our fits reproject 13% closer than MoSh++ (8.72 vs. 9.83 px), and MoSh++ is the worse of the two on 70% of all hands. As a body-wide fitting residual, we additionally report, for every (cleaned) VICON marker, the shortest distance to the fitted body surface—the GHUM mesh for our method, and the SMPL-X mesh that MoSh++ outputs for MoSh++. Our surface lies 8.49 mm from the markers on average (median 6.0) against 10.53 mm for MoSh++ (median 9.9), with the largest differences at the extremities: our fits place the ankles and toes within 3–5 mm of the markers, versus 12–14 mm for MoSh++. MoSh++ models each marker as sitting at a fixed learned offset off the body surface, so part of this gap reflects that modeling choice rather than fitting error; the extremity differences, however, exceed any uniform offset and follow from the hands and feet being left in less accurate configurations.

## 4. Evaluation Protocol and Benchmark

To help improve the state of the art in 3D human reconstruction for fitness training, we introduce a public challenge on the test set of *Fit3D*. We standardize the evaluation protocol and metrics to facilitate the comparison of different research methods.

Our evaluation server accepts multiple formats for the predicted reconstructions: GHUM [12] parameters, SMPL-X [13] parameters, and 3D joints in the Human3.6M format with 17 joints. The released test set contains only one random camera viewpoint per fitness sequence, so multi-view triangulation in reconstruction is not possible. Each prediction has to be provided in the coordinate system of the released camera. Predictions are required only on a subset of frames, equally spaced through each test sequence.

We report the following metrics, averaged over the sampled test frames:

- **MPJPE**: the mean per-joint position error, with and without Procrustes alignment, computed on the Human3.6M 17-joint configuration.
- **MPVPE**: the mean per-vertex position error, with and without Procrustes alignment, computed on the SMPL-X mesh when the submission provides SMPL-X parameters and on the GHUM mesh when it provides GHUM parameters.
- **MPVPE-T** (Mean Per-Vertex Position Error, T-pose): the predicted and reference bodies are posed in the same neutral T-pose using only their shape (β) parameters, and the distance between corresponding mesh vertices is averaged. Measures body-shape accuracy with pose removed.
- **MPJAE** (Mean Per-Joint Angle Error) [6]: for each SMPL-X body joint, the geodesic angle between the predicted and reference orientation (the smallest rotation aligning one to the other), averaged over joints. Measures rotation/pose accuracy, independent of limb lengths. We report it both without alignment (including overall body facing) and in its Procrustes-aligned form (PA-MPJAE), which first rotates the whole predicted body to best align it with the reference and so scores the articulated pose alone.
- **Translation error**: the $L_{2}$ distance between the predicted and reference root translation in the camera coordinate system.

Table 3 reports the performance of several recent monocular methods on the *Fit3D* test set. Every number in the table comes from our own runs: none are copied from the originating papers. For each of the 19 methods, we obtain its publicly available open-source implementation, run it ourselves on the *Fit3D* test frames under a single common protocol, and submit the resulting predictions to the evaluation server, which computes the reported metrics. Running every method ourselves—rather than collating self-reported numbers—is what makes the comparison controlled: all methods receive identical input frames, are evaluated on the same sampled test set, and are scored under the same metric definitions. When a method outputs only SMPL predictions, we convert them to SMPL-X before computing the vertex metrics; this conversion carries a mean vertex-to-vertex residual of approximately 3–5 mm, which slightly penalizes SMPL-output methods on MPVPE relative to methods that predict SMPL-X natively. REMIPS predicts GHUM natively; we submit its predictions in both GHUM and SMPL-X formats and, for comparability with the other rows, report its SMPL-X evaluation in the table.

Newer learned methods substantially improve over earlier optimization- and regression-based approaches: NLF [2] lowers the MPJPE from 119.45 mm (REMIPS) to 59.81 mm and the SMPL-X MPVPE-PA to 25.78 mm. The *Fit3D* training set has since been adopted by several recent large-scale methods: as reflected in the *Trained on Fit3D* column, the two methods that include *Fit3D* in their training data lead most reported metrics: NLF attains the lowest joint-position and vertex-position errors (and the lowest translation error among the methods evaluated under the true *Fit3D* intrinsics), while SMPLest-X attains the lowest joint-orientation errors; only the body-shape metric (MPVPE-T) is led by a method not trained on *Fit3D* (CameraHMR, by a 2 mm margin over SMPLest-X). This correlation should be read with care, however, NLF and SMPLest-X also differ from the other entries in architecture and in the remainder of their training corpora—NLF in particular leads several benchmarks it was not trained on—so the benchmark alone cannot attribute their lead to *Fit3D*. The controlled fine-tuning experiment of Section 6 isolates the contribution of *Fit3D* supervision from these confounding factors. Regardless of attribution, reconstructing fitness motions remains challenging, with the best non-aligned per-joint error still close to 60 mm, reflecting the out-of-distribution poses present in *Fit3D*.

Translation error varies by orders of magnitude across methods. For most of the benchmarked methods we obtain the root translation under the true *Fit3D* calibration—passing the intrinsics to the method where its interface accepts them (e.g., NLF, CameraHMR, GVHMR, SAM 3D Body), or re-solving the translation from the method’s image-aligned outputs under the true camera where it does not (e.g., 4DHumans, PARE, HybrIK); the resulting errors cluster at 185–350 mm (SMPLify-X excepted), reflecting the inherent monocular depth ambiguity, which grows on bent and ground-level poses. The remaining methods recover depth under their own camera model—a fixed 5000-pixel crop focal length for SMPLest-X, a self-estimated focal length for BLADE and PromptHMR, an image-diagonal focal length for WHAM and CLIFF—so their translation error measures the mismatch between assumed and true intrinsics rather than reconstruction quality: SMPLest-X places the body roughly an order of magnitude too deep, while CRMH predicts a near-zero root translation, so its error is dominated by the subject’s distance from the camera.

The benchmark, data, and submission instructions are publicly available at https://fit3d.imar.ro/ (accessed on 5 July 2026).

## 5. Analysis: Where Current Methods Succeed and Fail on *Fit3D*

Beyond the aggregate ranking of Table 3, the complementary metrics and a per-sequence breakdown over the 141 test sequences (3 subjects × 47 exercises) reveal *where* current methods break and what a fitness-capable reconstructor must improve.

No single method wins on all axes.

Position, orientation, and shape accuracy decouple. NLF attains the lowest joint- and vertex-*position* errors (MPJPE 59.81, PA-MPJPE 34.44, MPVPE-PA 25.78 mm), yet SMPLest-X is markedly the most accurate in joint *orientation* (MPJAE $9.32^{{}^{\circ}}$, PA-MPJAE $8.77^{{}^{\circ}}$, versus 12–$13^{{}^{\circ}}$ for NLF), and CameraHMR produces the most accurate body *shape* (MPVPE-T 23.61 mm). The ranking, therefore, reorders with the chosen axis, confirming that the orientation (MPJAE, PA-MPJAE) and shape (MPVPE-T) metrics capture failure modes that MPJPE alone does not: a method can localize joints well while misorienting limbs or defaulting to a generic body.

Most of the ranking is a statistical tie.

The differences near the top of Table 3 are smaller than the spread across sequences. A paired Wilcoxon signed-rank test on the per-sequence MPJPE shows that the six methods spanning 70.3–71.9 mm—CameraHMR, SAM 3D Body, TokenHMR, SMPLest-X, ScoreHMR, and GVHMR—are statistically indistinguishable from their immediate neighbors (p≥0.05 for each successive pair), whereas NLF is clearly separated from the field ($p<10^{-3}$). Sub-millimeter differences in aggregate MPJPE on *Fit3D* are, therefore, not meaningful in isolation; improvements should be demonstrated on the hard regime, where the spread is large.

Difficulty is strongly action-conditioned.

**Figure 4:**
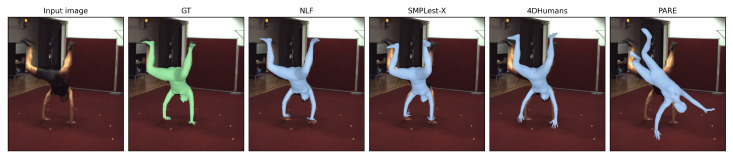
Qualitative reconstruction on a hard *Fit3D* pose (subject s02, *mule kick*—an inverted handstand-kick). From left: the input frame; the pseudo-ground-truth annotation and four benchmarked methods, each predicted SMPL-X mesh overlaid on the subject. To compare *pose and shape* independently of camera placement, all meshes are rendered at a common root depth: absolute placement is not comparable across methods because of the monocular depth–focal-length ambiguity, and is reported separately as the translation error. The *Fit3D*-trained methods (NLF, SMPLest-X) recover the inverted pose, while 4DHumans exhibits a large global-orientation error and PARE fails to recover the pose—the action-conditioned difficulty quantified in Table 4. The close agreement of the pseudo-ground-truth mesh with the subject also illustrates the quality of the released annotations.

Averaged over all methods, the hardest exercises incur roughly double the error of the easiest (Table 4). The hardest are dynamic, ground-level, and inverted poses with heavy self-occlusion—*mule kick*, *man maker*, *burpees*, *push-up*, and the forward-bend *warm-up 1* (118–155 mm). The easiest are upright, low-articulation exercises such as standing dumbbell curls and most other warm-up routines (∼67 mm). This gap persists even for the strongest method, and it is this out-of-distribution tail—not the near-upright exercises, which are largely solved—that future work should target. These are precisely the poses that the corpora dominating current training pipelines (Human3.6M, 3DPW, BEDLAM, AGORA) cover poorly. Figure 4 makes this concrete on an inverted *mule kick* frame: the *Fit3D*-trained NLF and SMPLest-X recover the handstand, whereas 4DHumans exhibits a large global-orientation error and the earlier PARE regressor fails to recover the pose.

Body shape is barely personalized.

The T-pose metric exposes that most methods cluster at MPVPE-T ≥30 mm: they recover a near-mean body rather than a subject-specific one, and even the best (CameraHMR, 23.6 mm) leaves a sizeable residual. This is an under-addressed axis. As Section 6 shows, adding *Fit3D* to the training mixture lowers HMR2.0’s MPVPE-T from 47.4 to 38.4 mm (Table 5), i.e., the network begins to predict the released per-subject shapes instead of defaulting to the mean. This gain comes less from additional pose data than from the in-domain camera intrinsics used in our supervision: because the *Fit3D* annotations are metric under the true focal length, training on them removes the depth–scale ambiguity and lets the network estimate translation and body shape more accurately.

The residual error is genuine, not only a camera artifact.

As noted above, absolute root translation is comparable only among the methods that consume the true intrinsics and is dominated by internal camera assumptions for the rest. Crucially, however, the error does not vanish once placement is removed: across the learned methods, PA-MPJPE remains 34–68 mm and PA-MPJAE 9–$19^{{}^{\circ}}$, so even after a global rigid alignment, a substantial articulation-and-shape gap persists. *Fit3D* releases per-sequence camera calibration, and a clear direction is for methods to consume it and produce metrically placed reconstructions rather than presuming a fixed focal length.

Takeaways for future methods.

Three priorities emerge. (i) *Personalized body shape*: most regressors default to a mean body, so subject-specific shape estimation is largely open. (ii) *The hard-pose tail*: ground-level, inverted, weight-loaded, and self-occluded articulations, where errors roughly double. (iii) *Metric placement under known intrinsics*. We further note that the classical optimization baseline (SMPLify-X, MPJPE 193 mm) trails the learned regressors by a wide margin under these out-of-distribution poses, and that the two methods trained with *Fit3D* (NLF and SMPLest-X) lead the position and orientation axes, respectively—underscoring in-domain data as the strongest available lever. Finally, because the released protocol samples sparse, equally spaced frames, it does not reward temporal consistency; this is why the video-based methods (GVHMR, WHAM, PromptHMR) are not advantaged here, and a dense-frame temporal track is a natural extension of the benchmark.

## 6. Impact of Fit3D Training: Fine-Tuning HMR2.0

The benchmark in Section 4 suggests that methods that include *Fit3D* in their training corpus achieve the lowest position and orientation errors. To isolate the effect of adding *Fit3D* from confounding factors such as differences in architecture and other training data, we run a controlled fine-tuning experiment, evaluating the resulting checkpoints both on the *Fit3D* test set (Table 5) and on AIST++ [54] as an out-of-domain generalization test on street dance motions (Table 6).

Fine-tuning setup.

We initialize from the public 4DHumans/HMR2.0 [1] model (ViT-H backbone) and fine-tune it on *Fit3D-train* mixed with the original HMR2.0 training corpus. To prevent catastrophic forgetting, *Fit3D* is given weight 0.3 in the sampling mixture and the original datasets (Human3.6M, COCO, MPII, AI-Challenger, AVA, MPI-INF-3DHP, InstaVariety) share the remaining 0.7; the adversarial body-pose prior of HMR2.0 is retained. We optimize with AdamW (learning rate $10^{-5}$, weight decay $10^{-4}$, mixed precision) and an effective batch of 256 (64 per GPU on 4 GPUs), evaluating the checkpoints at 30k, 60k, and 90k steps. Since *Fit3D* provides SMPL-X pseudo-ground-truth whereas HMR2.0 is trained on SMPL, we convert each frame’s SMPL-X fit to SMPL with the optimization-based transfer code provided by SMPL-X [13]—the same procedure we use to convert our GHUM fits to SMPL-X when building the annotations, after registering the topology of the two meshes; *Fit3D-train* is sub-sampled to 5 fps, yielding ∼178k images over its 8 subjects × 47 actions × 4 camera views.

On the *Fit3D* test set, every metric improves substantially after fine-tuning. MPVPE-T drops from 47.4 to 38.4 mm, indicating that the network learns to predict per-subject body shape parameters β that match the reference shapes released with our annotations, rather than defaulting to the mean shape produced by the released checkpoint. MPJAE drops from $16.1^{{}^{\circ}}$ to $10.4^{{}^{\circ}}$ and PA-MPJAE from $15.2^{{}^{\circ}}$ to $10.1^{{}^{\circ}}$, a relative reduction of approximately 33%, tracking the gains in joint and vertex position errors. The improvements largely saturate by 30k–60k steps, with the 90k checkpoint offering only marginal additional gains.

The gains concentrate on the hard poses.

**Figure 5:**
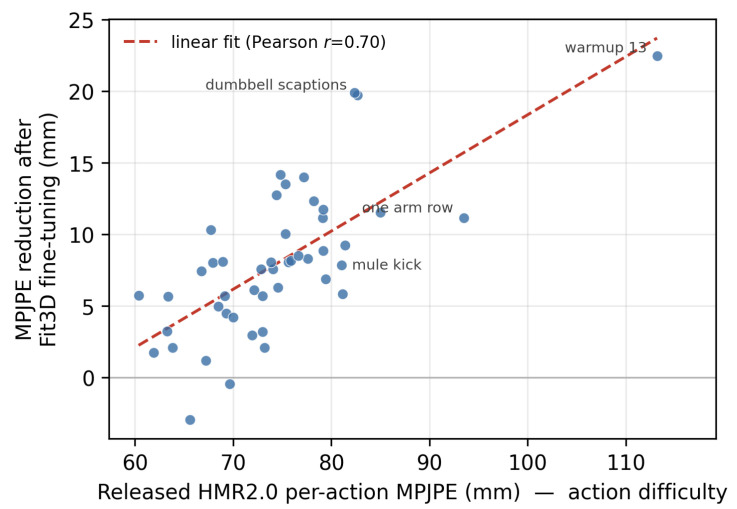
Fine-tuning gains concentrate on the hard actions. Each point is one of the 47 *Fit3D* exercises; the *x*-axis is the released HMR2.0 per-action MPJPE (a proxy for difficulty) and the *y*-axis is the per-action MPJPE reduction after fine-tuning on *Fit3D-train* (60k steps). The improvement grows with difficulty (Pearson r=0.70): the hardest exercises gain the most, mirroring the action-conditioned difficulty of Section 5.

Section 5 showed that errors on *Fit3D* are strongly action-conditioned. Breaking the fine-tuning improvement down per exercise (Figure 5) shows that it is exactly the hard actions that benefit most: the per-action MPJPE reduction is positively correlated with the released model’s per-action error (Pearson r=0.70), and the ten hardest exercises improve by 12.3 mm on average against only 4.2 mm for the ten easiest. In other words, *Fit3D* supervision helps precisely where current methods fail—on the out-of-distribution, heavily articulated poses that the standard training corpora do not cover—rather than uniformly shifting an already-well-reconstructed regime.

On AIST++, all eight metrics remain within noise across fine-tuning checkpoints—MPJAE stays around $23^{{}^{\circ}}$ and PA-MPJAE around $21.5^{{}^{\circ}}$ throughout—confirming that fine-tuning on *Fit3D* does not degrade out-of-domain generalization. Two cross-dataset caveats apply: (†) MPVPE-T on AIST++ measures only the deviation of the predicted shape from neutral, because AIST++ ground truth uses β=0, so the column is not directly comparable with the *Fit3D* MPVPE-T column and MPVPE / MPVPE-PA on AIST++ are computed against SMPL ground truth, whereas on *Fit3D* they are computed against SMPL-X.

## 7. Discussion

The benchmark and the fine-tuning study together point to in-domain supervision as the strongest available lever for fitness-domain reconstruction, with personalized body shape, the hard-pose tail, and metric placement under known intrinsics as the main open axes (Section 5). At the same time, four caveats bound the contributions of this work.

First, the released GHUM/SMPL-X annotations are pseudo-ground-truth obtained by optimization rather than by direct measurement: the loss weights $\lambda_{•}$ were hand-tuned, the body prior was down-weighted per sequence on out-of-distribution motions, and the fits were validated by multi-view visual inspection together with the marker-to-surface residual (8.49 mm mean, Section 3.3) rather than against an independent sensor, so a small residual error on the most extreme poses cannot be excluded. Second, the hand-articulation comparison of Section 3.3 scores each fit against 2D keypoint detections, the same modality our pipeline optimizes through $L_{2D}$ (whereas MoSh++ ignores it); it should, therefore, be read as a consistency check that favors multi-view-supervised fits over marker-only fits, not as fully independent validation. Third, *Fit3D* is a single-person, indoor laboratory capture of 13 subjects in tight gym attire under controlled studio lighting and fixed calibrated cameras; multi-person interaction, outdoor and truly in-the-wild imagery, loose or varied clothing, uncontrolled capture conditions, and a broader demographic range lie outside its scope and outside what the benchmark currently measures, so the generalization of models trained on these annotations to real-world fitness videos remains to be established. Because the train/validation/test split is subject-disjoint, our benchmark and fine-tuning results already measure generalization to unseen *subjects*, but not across these other axes of variation. Fourth, the benchmark protocol scores sparsely sampled frames independently, which is fair to image-based methods but does not credit the temporal consistency that video-based methods can provide; adding temporal-consistency metrics (e.g., acceleration error) and dense per-sequence evaluation is a natural extension for future versions of the benchmark.

## 8. Conclusions

Building on the *Fit3D* fitness dataset of Fieraru et al. [11], this paper has contributed three extensions for the monocular 3D human reconstruction community. First, we have described the methodology used to construct dense GHUM and SMPL-X pseudo-ground-truth shape and pose annotations on top of the raw Fit3D MoCap, a marker- and multi-view-keypoint-driven fitting pipeline regularized by separate body and hand normalizing-flow priors and a self-collision loss, designed to remain stable under the extreme articulations and self-occlusions characteristic of fitness motions. Second, we have defined a standardized evaluation protocol—metric set, frame sampling, and coordinate conventions, served through the IMAR-hosted Fit3D evaluation server—and conducted a comparative benchmark study of 19 representative methods spanning optimization-based, single-frame, and video-based families, finding that the two methods that include *Fit3D* in their training corpus lead the position and orientation metrics, respectively. Third, a controlled fine-tuning experiment showed that adding *Fit3D* to the training mixture of a strong baseline improves it most on exactly the hard, out-of-distribution poses where current methods fail, without regressing out-of-domain performance—direct evidence that in-domain supervision is the strongest available lever. The *Fit3D* dataset and the GHUM/SMPL-X annotations are available, under a non-commercial research license, through the IMAR Fit3D resource [11] at https://fit3d.imar.ro/ (accessed on 5 July 2026); to date (June 2026), 1088 academics have registered for access, indicating broad interest in the dataset and benchmark.

## Figures and Tables

**Table 1:** *Fit3D* in the context of 3D human pose and shape datasets. With released GHUM/SMPL-X annotations on a fitness corpus, *Fit3D* is the only dataset combining high-fidelity marker MoCap, multi-view RGB, parametric whole-body pseudo-ground-truth, and a fitness focus. In the Hands column,  ✓ indicates that articulated hand annotations are provided and – that they are not.

Dataset	Subjects	Frames	GT Source	Cameras	Body-Model GT	Hands	Setting/Focus
Human3.6M [8]	11	3.6 M	optical marker MoCap	4 (+10 mocap)	3D joints	–	indoor lab/daily activities
MPI-INF-3DHP [18]	8	1.3 M	markerless multi-view MoCap	14	3D joints	–	studio + outdoor/general poses
3DPW [6]	7	51 k	IMU + handheld video	1 moving	SMPL	–	in-the-wild/daily motion
EMDB [7]	10	∼100 k	electromagnetic sensors	1 moving	SMPL	–	in-the-wild/world-grounded motion
RICH [32]	22	577 k	markerless MoCap + scene scans	6–8	SMPL-X	✓	indoor + outdoor/ human–scene contact
MOYO [33]	1	1.75 M	marker MoCap + pressure mat	8	SMPL-X	✓	indoor lab/yoga, extreme poses
BEDLAM [9]	271	380 k	synthetic render	synthetic	SMPL-X	✓	synthetic/appearance diversity
AGORA [10]	4240	17 k	synthetic render of 3D scans	synthetic	SMPL & SMPL-X	✓	synthetic/ multi-person, occlusion
*Fit3D* (ours) [11]	13	3.0 M	marker MoCap + multi-view RGB + scans	4 (+12 mocap)	GHUM & SMPL-X	✓	indoor lab/fitness exercises

**Table 2:** Quantitative comparison with MoSh++ on the eight *Fit3D* training subjects. *Hand reproj.*: mean distance (px) between projected 3D hand joints and detected 2D hand keypoints over the four RGB views. *Marker–surf.*: mean shortest distance (mm) from each (cleaned) VICON marker to the fitted body mesh. Lower is better (indicated by ↓); best in **bold**.

	Hand Reproj. (px) ↓	Marker–Surf. (mm) ↓
MoSh++ [51]	9.83	10.53
Ours	8.72	8.49

**Table 3:** Benchmark of monocular 3D human reconstruction methods on the *Fit3D* test set, sorted by MPJPE (best first). All position errors are in millimeters and angle errors (MPJAE, PA-MPJAE) in degrees. ^†^ Translation reported under the method’s own camera model (a fixed or self-estimated focal length) rather than the true *Fit3D* intrinsics; these values measure camera-assumption mismatch and are not comparable with the other rows (see text). * NLF predicts a non-parametric Neural Localizer Field; we evaluate using its SMPL-X regression head. Lower is better for all metrics (indicated by ↓); the best value in each column is in **bold**.

Method	Year	Trained on Fit3D	Input	Output	Transl. Err. ↓	3D Joints		SMPL-X				
						MPJPE ↓	MPJPE-PA ↓	MPVPE ↓	MPVPE-PA ↓	MPVPE-T ↓	MPJAE ↓	PA-MPJAE ↓
NLF [2]	2024	Yes	Image	SMPL-X *	185.16	59.81	34.44	51.41	25.78	31.31	12.83	12.59
CameraHMR [3]	2025	No	Image	SMPL	212.29	70.31	39.69	59.80	32.64	23.61	14.87	14.31
SAM 3D Body [5]	2026	No	Image	MHR	187.67	70.65	37.89	56.74	28.89	29.13	12.62	12.34
TokenHMR [38]	2024	No	Image	SMPL	301.33	71.21	37.05	72.28	36.91	37.50	15.80	14.46
SMPLest-X [4]	2025	Yes	Image	SMPL-X	${34,668.77\;}^{†}$	71.51	40.35	54.60	26.86	25.66	9.32	8.77
ScoreHMR [44]	2024	No	Image	SMPL	332.48	71.61	37.00	73.98	34.18	46.94	14.71	13.90
GVHMR [42]	2024	No	Video	SMPL-X	249.72	71.94	40.07	64.62	35.57	35.22	14.76	14.25
4DHumans [1]	2023	No	Image	SMPL	337.33	74.48	37.56	77.26	38.37	47.43	16.14	15.16
PromptHMR [43]	2025	No	Video	SMPL-X	${1078.62\;}^{†}$	75.09	42.51	62.17	32.79	23.71	13.51	12.85
WHAM [41]	2024	No	Video	SMPL	${931.43\;}^{†}$	85.85	46.74	84.99	41.76	42.48	18.58	17.26
Multi-HMR [39]	2024	No	Image	SMPL-X	342.65	89.16	50.07	77.71	39.82	34.42	15.66	14.66
PyMAF-X [36]	2023	No	Image	SMPL-X	350.05	89.60	48.61	74.79	38.74	42.42	15.82	14.82
PARE [34]	2021	No	Image	SMPL	296.86	89.90	53.74	85.10	46.84	35.65	18.33	16.47
BLADE [40]	2025	No	Image	SMPL-X	${2374.31\;}^{†}$	95.19	55.34	83.05	46.67	34.28	17.06	15.53
HybrIK [35]	2021	No	Image	SMPL-X	322.85	97.56	50.76	91.45	41.60	36.12	18.44	17.16
CLIFF [37]	2022	No	Image	SMPL	${896.49\;}^{†}$	105.42	55.17	107.73	51.54	48.25	20.80	16.24
CRMH [53]	2020	No	Image	SMPL	${3283.89\;}^{†}$	116.19	62.41	120.12	59.86	56.52	23.53	18.52
REMIPS [48]	2021	No	Image	GHUM	667.01	119.45	67.51	112.53	58.68	39.52	19.38	16.77
SMPLify-X [13]	2019	No	Image	SMPL-X	1388.61	193.16	94.88	197.09	88.88	92.02	33.49	25.41

**Table 4:** Hardest and easiest *Fit3D* exercises, mean error over all 19 benchmarked methods and the 3 test subjects. Position errors in mm, MPJAE in degrees. The italicized rows are group labels separating the five hardest from the five easiest exercises.

Exercise	MPJPE	MPJPE-PA	MPJAE
*Hardest*			
mule kick	155.5	71.2	28.7
man maker	135.3	73.1	22.6
burpees	121.9	62.1	20.7
push-up	118.7	61.2	21.6
warm-up 1	118.0	61.6	19.2
*Easiest*			
dumbbell biceps curls	71.5	39.3	15.6
warm-up 6	69.9	35.2	12.1
warm-up 14	69.1	39.1	14.9
warm-up 8	67.6	35.5	12.0
dumbbell hammer curls	67.0	35.6	12.5

**Table 5:** *Fit3D* test set: released 4D-Humans (HMR2.0) vs. fine-tuning on *Fit3D-train* (30k/60k/90k steps), all 141 test sequences. MPJPE/MPJPE-PA on joints3d (H36M-17); MPVPE/MPVPE-PA on posed SMPL-X meshes; MPVPE-T on the T-pose (shape only); MPJAE/PA-MPJAE on SMPL-X body-joint orientations. Lower is better; best in **bold**.

	Transl. (mm)	MPJPE (mm)	MPJPE-PA (mm)	MPVPE (mm)	MPVPE-PA (mm)	MPVPE-T (mm)	MPJAE (°)	PA-MPJAE (°)
Released (HMR2.0)	337.3	74.5	37.6	77.3	38.4	47.4	16.1	15.2
+ FT (30k)	243.5	66.2	35.9	53.3	24.3	38.4	10.8	10.4
+ FT (60k)	236.9	66.5	36.2	55.9	25.2	37.8	10.6	10.2
+ FT (90k)	249.7	66.7	36.0	56.0	24.3	39.4	10.4	10.1

**Table 6:** AIST++ (out-of-domain street dance): generalization after *Fit3D* fine-tuning (30k/60k/90k steps). Joint metrics over 19 test sequences; mesh and angle metrics over the eight sequences with ground-truth SMPL. All differences are within noise—fine-tuning does not regress out-of-domain performance. Lower is better. ^†^ MPVPE-T on AIST++ measures only the deviation of the predicted shape from the neutral body, because the AIST++ ground truth uses β=0; it is therefore not comparable with the *Fit3D* MPVPE-T column (see text).

	Transl. (mm)	MPJPE (mm)	MPJPE-PA (mm)	MPVPE (mm)	MPVPE-PA (mm)	MPVPE-T ^†^ (mm)	MPJAE (°)	PA-MPJAE (°)
Released (HMR2.0)	501.2	164.1	108.9	109.0	69.2	22.6	23.0	21.6
+ FT (30k)	516.0	165.1	107.4	110.2	68.3	24.0	22.5	21.0
+ FT (60k)	511.0	163.6	107.7	110.8	69.1	20.9	22.6	21.1
+ FT (90k)	496.3	164.0	107.9	110.7	68.8	21.9	22.7	21.2

## Data Availability

Restrictions apply to the availability of these data. Data were obtained from the Institute of Mathematics of the Romanian Academy (IMAR) and are available at https://fit3d.imar.ro/ (accessed on 5 July 2026) [11] with the permission of IMAR. The *Fit3D* dataset is the property of the Institute of Mathematics of the Romanian Academy (IMAR) and is available under a non-commercial research license. The GHUM/SMPL-X annotations and the evaluation protocol described in this paper are accessible through that same resource, subject to that license. Users of the dataset and annotations are required to cite the original *Fit3D* publication [11].
